# Significant Performance Improvement in n‐Channel Organic Field‐Effect Transistors with C_60_:C_70_ Co‐Crystals Induced by Poly(2‐ethyl‐2‐oxazoline) Nanodots

**DOI:** 10.1002/adma.202100421

**Published:** 2021-06-24

**Authors:** Sungho Nam, Dongyoon Khim, Gerardo T. Martinez, Aakash Varambhia, Peter D. Nellist, Youngkyoo Kim, Thomas D. Anthopoulos, Donal D. C. Bradley

**Affiliations:** ^1^ Department of Physics University of Oxford Oxford OX1 3PD UK; ^2^ Blackett Laboratory Department of Physics and Centre for Plastic Electronics Imperial College London London SW7 2BW UK; ^3^ Department of Materials University of Oxford Oxford OX1 3PH UK; ^4^ Organic Nanoelectronics Laboratory and KNU Institute for Nanophotonics Applications (KINPA) School of Applied Chemical Engineering Kyungpook National University Daegu 41566 Republic of Korea; ^5^ Physical Science and Engineering Division King Abdullah University of Science and Technology (KAUST) Thuwal 23955‐6900 Saudi Arabia

**Keywords:** C60:C70 co‐crystals, nanodot layers, n‐channel organic field‐effect transistors, poly(2‐ethyl‐2‐oxazoline)

## Abstract

Solution‐processed organic field‐effect transistors (OFETs) have attracted great interest due to their potential as logic devices for bendable and flexible electronics. In relation to n‐channel structures, soluble fullerene semiconductors have been widely studied. However, they have not yet met the essential requirements for commercialization, primarily because of low charge carrier mobility, immature large‐scale fabrication processes, and insufficient long‐term operational stability. Interfacial engineering of the carrier‐injecting source/drain (S/D) electrodes has been proposed as an effective approach to improve charge injection, leading also to overall improved device characteristics. Here, it is demonstrated that a non‐conjugated neutral dipolar polymer, poly(2‐ethyl‐2‐oxazoline) (PEOz), formed as a nanodot structure on the S/D electrodes, enhances electron mobility in n‐channel OFETs using a range of soluble fullerenes. Overall performance is especially notable for (C_60_‐I_h_)[5,6]fullerene (C_60_) and (C_70_‐D_5h(6)_)[5,6]fullerene (C_70_) blend films, with an increase from 0.1 to 2.1 cm^2^ V^−1^ s^−1^. The high relative mobility and eighteen‐fold improvement are attributed not only to the anticipated reduction in S/D electrode work function but also to the beneficial effects of PEOz on the formation of a face‐centered‐cubic C_60_:C_70_ co‐crystal structure within the blend films.

## Graphical abstract

18‐fold‐enhanced electron mobility is achieved for n‐channel organic field‐effect transistors with co‐crystalized C_60_:C_70_ blend films formed on nanodot‐structured dipolar poly(2‐ethyl‐2‐oxazoline) (PEOz) interlayers that also reduce the work function of the electrodes. The PEOz nanodot layers further deliver high spatial uniformity in electron mobility and enhance device stability for transistor array fabrication.

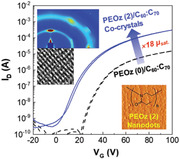

## 1 Introduction

With the development of novel organic semiconductor materials and printing techniques, the interest in printed organic electronics has grown tremendously in recent years due to its great potential for use in the emerging Internet of Things (IoT) device ecosystem where multiple device nodes share information with cloud networking systems.^[^ ^1^, ^2^, ^3^, ^4^ ^]^ Printed organic electronics can enable the mass production and integration of electronic functionalities using novel substrates.^[^ ^5^, ^6^, ^7^, ^8^, ^9^, ^10^ ^]^ In particular, organic field‐effect transistors (OFETs) are regarded as a key electronic element in integrated logic circuits found at the heart of IoT.^[^ ^11^, ^12^ ^]^ High‐performance OFETs with low‐voltage operation (<5 V) and charge carrier mobility beyond that of amorphous silicon (0.5–1 cm^2^ V^–1^ s^–1^) have been demonstrated.^[^ ^13^, ^14^, ^15^, ^16^, ^17^, ^18^, ^19^, ^20^, ^21^ ^]^ Successful commercialization additionally requires spatially uniform device performance across the many devices on a substrate, including reproducibility in characteristics and environmental stability.

In general, device performance is largely determined by material properties and the interfaces between the electrodes, dielectric, and semiconductor. A variety of strategies to improve OFET performance have been reported, such as development of new materials via rational molecular design, morphology control through thermal/solvent annealing and additives, molecular doping with p‐/n‐dopants, and interfacial engineering.^[^ ^7^, ^15^, ^18^, ^22^, ^23^, ^24^ ^]^ Of these approaches, interfacial engineering of the source–drain (S–D) electrodes and organic semiconductor layer through the use of organic dipole interlayers is of great importance in determining the charge injection and extraction efficiency.^[^ ^25^, ^26^, ^27^ ^]^ To date, various interlayer materials have been proposed to reduce the energy barrier between the metal electrode and organic semiconductors in n‐type OFETs, including neutral dipolar and ionic (polyelectrolyte) polymers, alkali metal salts, self‐assembled dipole monolayers, and metal‐oxide and metal‐carbonate thin films.^[^ ^26^, ^27^, ^28^, ^29^, ^30^, ^31^, ^32^, ^33^ ^]^

Among them, non‐conjugated neutral dipolar poly(2‐alkyl‐2‐oxazoline) (PAOz) polymers have intriguing properties, including synthetic versatility with good biocompatibility and diverse functionalities.^[^ ^34^, ^35^, ^36^, ^37^, ^38^ ^]^ We recently reported the improved performance of polymer solar cells using distinctive poly(2‐ethyl‐2‐oxazoline) (PEOz) nanodot and nanocrater structures formed on electron‐collecting buffer layers.^[^ ^39^, ^40^ ^]^ Subsequently, the effect on OFET performance of different alkyl side chains in PAOz was also examined.^[^ ^41^ ^]^ PEOz polymers have also been incorporated in perovskite solar cells and light‐emitting diodes to enhance device performance.^[^ ^42^, ^43^, ^44^ ^]^ However, despite the suitability of PEOz polymers for tailoring the efficiency of numerous devices, the effect of the PEOz nanodot layer on the surface morphology/nanostructure of the overlying organic semiconductor has not yet been thoroughly investigated.

In this study, PEOz nanodot layers are used to enhance the performance of solution‐processed fullerene n‐type OFETs. PEOz films were spin‐coated from different solution concentrations in methanol and the impact on transistor performance was studied for two additonal polymer semiconductors, poly([*N*,*N*′‐bis(2‐octyldodecyl)‐naphthalene‐1,4,5,8‐bis(dicarboximide)‐2,6‐diyl]‐*alt*‐5,5′‐(2,2′‐bithiophene) (P(NDI2OD‐T2)) and poly[[2,5‐bis(2‐octyldodecyl)‐2,3,5,6‐tetrahydro‐3,6‐dioxopyrrolo[3,4‐c]pyrrole‐1,4‐diyl]‐*alt*‐[[2,2′‐(2,5‐thiophene)bis‐thieno(3,2‐b)thiophene]‐5,5′‐diyl]] (P(DPPT‐TT)). The impressive results for C_60_:C_70_ fullerene blends are presented in full within the manuscript, with results for the other soluble fullerenes and polymers covered in Supporting Information. Table S1, Supporting Information summarizes the OFET data for all semiconductors studied. Ultraviolet photoelectron spectroscopy (UPS) and Kelvin probe (KP) measurements were performed to examine the work function change for S–D electrodes coated with a PEOz interlayer and PEOz nanodot structures were characterized using atomic force microscopy (AFM). The surface morphology and nanostructure of C_60_:C_70_ fullerene blend films were additionally investigated by AFM, grazing‐incidence X‐ray diffraction (GIXD), and high‐resolution transmission electron microscopy (HR‐TEM). For all of the studied semiconductors the use of PEOz yielded enhanced performance but this was especially so for OFETs with C_60_:C_70_ fullerene blend films deposited on PEOz spin‐coated from 2 mg mL^−1^ methanol solution where an ≈18.6‐fold increase in field‐effect mobility from 0.11 to 2.1 cm^2^ V^−1^ s^−1^ was achieved. The particular influence of the PEOz interlayer on the device performance in this case was clarified by comparison with the data for the other soluble fullerene and polymer semiconductors, showing a strong effect of the blend film microstructure. Spatial uniformity of device performance was subsequently tested for batches of 40 C_60_:C_70_ fullerene blend devices configured as 10 × 4 arrays, both with and without the PEOz interlayer.

## 2 Results and Discussion

### 2.1 PEOz Deposition and Characterization

**Figure 1:**
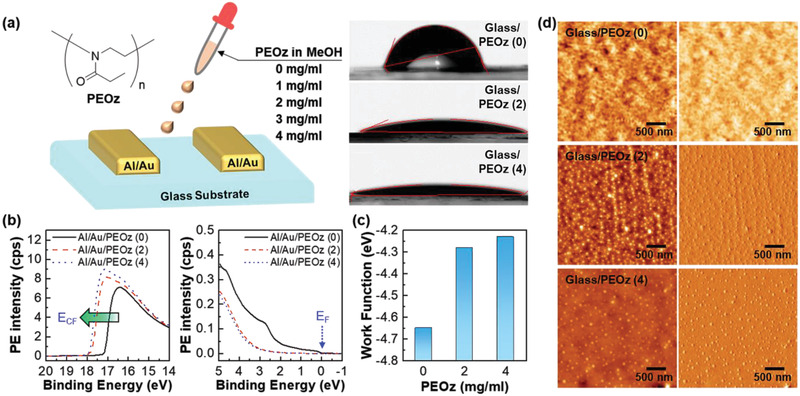
a) Chemical structure of PEOz polymer and schematic of PEOz coating on Al/Au‐electrode‐coated glass substrates. Water droplet contact angles are shown to the right for PEOz on bare glass substrates and substrates spin‐coated with PEOz (2) and PEOz (4) solutions. b) UPS spectra across two energy ranges showing the reduced work function (Φ) according to the following equation: Φ = *hν* − (*E*_CF_ − *E*_F_) where *hν* = 21.2 eV for He(I) and *E*_CF_ and *E*_F_ (indicated in the figures) are the secondary electron cut‐off and Fermi energies, respectively. c) KP work function values for the bare and PEOz‐coated Al/Au electrodes. d) Height‐mode (left column) and phase‐mode (right column) AFM images for bare and PEOz‐coated glass substrates. Note that PEOz (0), PEOz (2), and PEOz (4) refer to samples with no PEOz layer and with PEOz layers deposited from 2 and 4 mg mL^−1^ methanol solutions, respectively.

Bare and PEOz‐coated glass substrates were characterized by contact angle measurements, with the sample labels PEOz (0), PEOz (2), and PEOz (4) denoting, respectively, no PEOz and PEOz films spin‐coated from 2 and 4 mg mL^−1^ solution. As shown in **Figure** **1**a, the contact angles for water significantly reduced in the presence of the PEOz interlayer, yielding 68°, 22°, and 13° for PEOz (0), PEOz (2), and PEOz (4), respectively. The work function change of Al/Au S–D electrodes coated with a PEOz interlayer was studied via UPS measurements. Figure 1b shows the PEOz film UPS spectra, with a clear spectral shift toward higher binding energy in the cutoff region for films deposited from higher solution concentration. The electrode work function shifts from −4.06 eV to −3.37 eV for Al/Au/PEOz (2) and to −3.27 eV for Al/Au/PEOz (4). This trend was also confirmed by KP measurements (Figure 1c), with work function shifts of ≈ 0.37 and ≈ 0.42 eV for PEOz (2) and PEOz (4) films, respectively. The PEOz interlayer dipole reduces the Al/Au electrode work function. The difference in the work function values determined by UPS and KP measurements can be primarily attributed, as previously well‐documented, to the different measurement environments, namely in vacuo for UPS and in ambient for KP.^[^ ^45^, ^46^, ^47^ ^]^

AFM measurements were conducted on PEOz films spin‐coated on glass substrates and on top of Al/Au electrodes. Figure 1d shows randomly distributed nanodots across the entire surface for glass/PEOz (2) and glass/PEOz (4) samples, with diameters ≈20–50 nm and 50–120 nm, respectively. Both glass/PEOz (2) and glass/PEOz (4) films have a relatively smooth surface with root‐mean‐square roughnesses (R_RMS_) of only 0.50 and 0.49 nm, respectively. The relatively rough surface of the bare Al/Au electrode (*R*_RMS_ = 3.69 nm) was significantly smoothed by the PEOz‐coating, giving *R*_RMS_ = 2.99 and 2.97 nm for Al/Au/PEOz (2) and Al/Au/PEOz (4), respectively (Figure S1, Supporting Information). This is anticipated to help reduce the energetic disorder in the organic semiconductor in the vicinity of the electrode. A conformal coverage of the Al/Au electrode for both PEOz interlayers (2 and 4 mg mL^−1^) was observed in phase‐mode AFM images, with the appearance of a grain‐like nanostructure. The combined work function and structural changes are expected to enhance charge injection and transport.

### 2.2 Transistor Fabrication and Characterization

**Figure 2:**
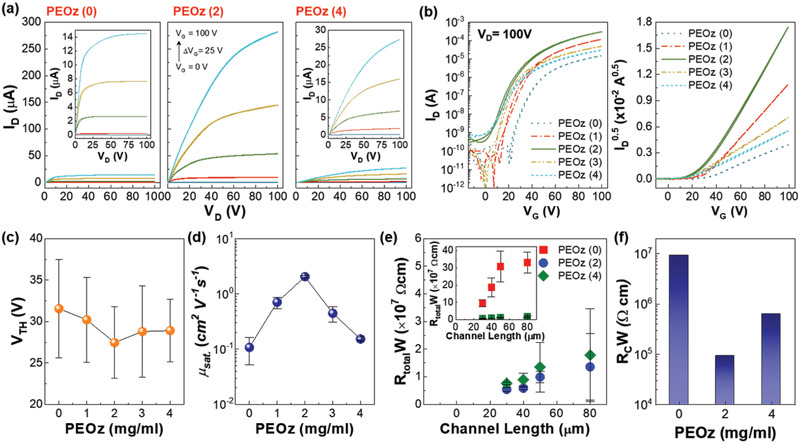
a) Output (*I*_D_–*V*_D_) and b) transfer (*I*_D_–*V*_G_) characteristics at *V*_D_ = 100 V for C_60_:C_70_ transistors fabricated using PEOz solution concentrations in the 0–4 mg mL^−1^ range. c) Threshold voltage (*V*_TH_) and d) field‐effect mobility in the saturation regime (μ_sat_) as a function of PEOz solution concentration. e) Total resistivity (*R*_total_ *W*) as a function of channel length and f) deduced contact resistivity (*R*_C_ *W*) determined by the transfer length method. Note that all transistor parameters were obtained for more than ten devices, and the values shown are averages with the error bars representing standard deviations.

Top‐gate, bottom‐contact, n‐type transistors were, therefore, fabricated to test the influence of PEOz interlayer films of different thicknesses on transistor performance. Films were deposited from 0 to 4 mg mL^−1^ solutions (see Experimental Section and the device structure in Figure S2, Supporting Information) and as shown in **Figure** **2**a and Figure S3, Supporting Information, the drain current (*I*_D_) in the output characteristics (*I*_D_ versus *V*_D_) for C_60_:C_70_ transistors was significantly influenced thereby. In particular, *I*_D_ for the C_60_:C_70_ transistor with a PEOz (2) interlayer at *V*_D_ = *V*_G_ = 100 V was drastically increased, ≈20‐fold compared to that with no PEOz interlayer. *I*_D_ then gradually decreased on increasing the PEOz solution concentration above 2 mg mL^−1^; the measured *I*_D_ values for PEOz (0), PEOz (2), and PEOz (4) transistors were 14, 282, and 27 µA, respectively. Furthermore, transfer curves (*I*_D_ versus *V*_G_) for C_60_:C_70_ transistors with a PEOz (2) interlayer showed a substantial increase in *I*_D_ together with an obvious shift toward lower voltages (Figure 2b). Increasing the solution concentration further, however, then results in a decrease in *I*_D._ The threshold voltage (*V*_TH_) shows similar non‐monotonic behavior, with a gradual decrease in *V*_TH_ up to 2 mg mL^−1^ followed by an increase for higher concentrations; the measured *V*_TH_ values for PEOz (0), PEOz (2), and PEOz (4) transistors were 32, 27, and 29 V, respectively (Figure 2c). The field‐effect mobility in the saturation region (μ_sat_) also showed a non‐monotonic variation with PEOz thickness. The highest mobility obtained was 2.1 cm^2^ V^−1^ s^−1^ for the PEOz (2) interlayer device and higher PEOz solution concentrations gave lower mobilities; μ_sat_ values of 0.11 and 0.15 cm^2^ V^−1^ s^−1^ were obtained for the PEOz (0) and PEOz (4) devices, respectively (Figure 2d). Note also that the C_60_:C_70_ blend PEOz (2) device shows higher μ_sat_ than corresponding PEOz (2) devices with either C_60_ or C_70_ on their own (see Figure S4, Supporting Information). The improved transistor performance correlates also with a reduced total resistivity, *R*_total_ *W*, where *R* is resistance and *W* channel length, and also the contact resistivity *R*_C_ *W* determined by extrapolating *R*_total_ *W* to zero channel length.^[^ ^41^, ^48^ ^]^ *R*_total_ *W* is lower for C_60_:C_70_ blend devices with a PEOz (2) interlayer than for those with either PEOz (0) or PEOz (4) interlayers (Figure 2e) and the deduced *R*_C_ *W* values of 9.3 × 10^6^ for PEOz (0), 9.3 × 10^4^ for PEOz (2), and 6.4 × 10^5^ for PEOz (4) interlayer devices mirror this (Figure 2f).

Operational stability was also investigated for C_60_:C_70_ PEOz (0) and PEOz (2) transistors held under constant bias at *V*_D_ = *V*_G_ = 30 V for 600 s (see Figure S5a, Supporting Information). Transfer curves for the device without a PEOz interlayer noticeably shift to higher voltage, whereas the PEOz (2) interlayer device is much less affected. The change in *V*_TH_ for the PEOz (0) device was Δ*V*_TH_ ≈ 11.1 V whilst Δ*V*_TH_ ≈ 1.8 V for the PEOz (2) device, suggesting substantially less charge carrier trapping at the electrode interface in the presence of the PEOz (2) interlayer. In addition, the C_60_:C_70_ PEOz (2) device has a long shelf‐life, showing little detrimental effect after 30 days glove‐box storage. Ambient operation is also relatively good with marginal degradation in terms of V_TH_ and μ_sat_ (see Figure S5b, Supporting Information).

The beneficial effect of PEOz interlayers on n‐type organic transistor performance was also investigated for soluble fullerene derivatives [6,6]‐phenyl‐C_61_‐butyric acid methyl ester (PC_61_BM), [6,6]‐phenyl‐C_71_‐butyric acid methyl ester (PC_71_BM), and indene‐C_60_ bisadduct (ICBA) and for the conjugated polymers P(NDI2OD‐T2) and P(DPPT‐TT). The optimal performance of PEOz (2) interlayers was confirmed for all, supported by the resulting superior transistor characteristics, in terms of V_TH_ and μ_sat_, relative to devices with either no PEOz or thicker PEOz interlayers (Figures S6–S9 and Table S1, Supporting Information). In the case of the fullerene derivatives PC_61_BM,PC_71_BM and ICBA and P(NDI2OD‐T2), the dependence of μ_sat_ is less clearly non‐monotonic with PEOz thickness. The mobility value shows instead a step rise on introduction of the PEOz interlayer and is then relatively static; it also remains more than an order of magnitude lower than for the corresponding C_60_:C_70_ blend devices.

### 2.3 C_60_:C_70_ Blend Film Microstructure

**Figure 3:**
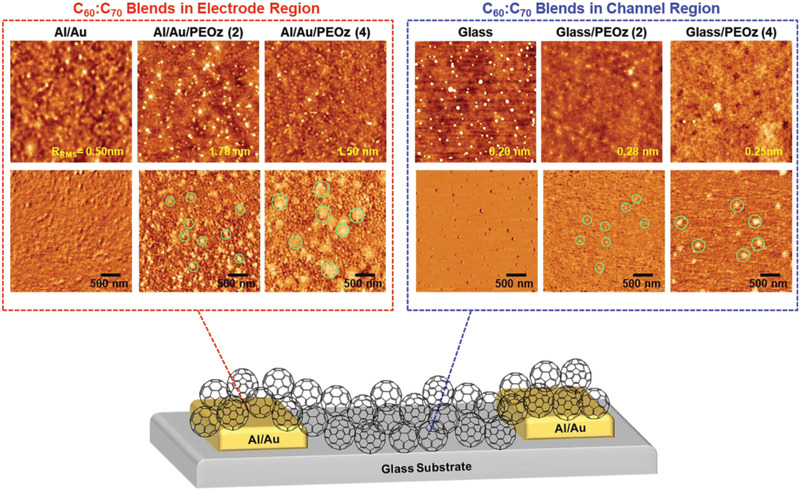
Height‐mode (upper row) and phase‐mode (lower row) AFM images for C_60_:C_70_ fullerene blend coated PEOz (0), PEOz (2), and PEOz (4) samples in the electrode (left red dashed box) and channel (right blue dashed box) regions.

The non‐monotonic behavior for μ_sat__t_ in C_60_:C_70_ transistors contrasts with the monotonic shift in work function (Figure 1c), indicating that effects other than just a reduction in energy barrier at the electrode–semiconductor interface must be at work. To investigate the potential influence of structural factors, the surface morphology of C_60_:C_70_ blend films within the electrode and channel regions was examined using AFM. As shown in **Figure** **3**, a smooth and homogenous surface was observed for the C_60_:C_70_ blend films on bare (without a PEOz interlayer) Al/Au, while in the channel region on bare glass, island‐like nanoaggregates were observed, possibly due to a partial dewetting. Charge carrier scattering (i.e., trapping) may therefore be expected at the interface between the electrode and interlayer‐induced grain boundaries within the channel region, resulting in lower μ_sat_. A pronounced change in the surface morphology was observed for the C_60_:C_70_ blend films on top of PEOz interlayers. In particular, the phase‐mode AFM images exhibit bright, roughly circular structures with nanometer diameters that are larger in the electrode regions and increase with PEOz solution concentration. Their diameters fall in the ranges 20–30, 50–100, 100–120, and 300–500 nm for C_60_:C_70_ blend films on glass/PEOz (2), Al/Au/PEOz (2), glass/PEOz (4), and Al/Au/PEOz (4), respectively. The phase shift structures can be attributed to the presence of the PEOz interlayer underneath the C_60_:C_70_ blend layer. The bright and dark regions are expected to strongly correlate with the surface stiffness/softness and adhesion between the AFM tip and sample surface.^[^ ^49^, ^50^, ^51^ ^]^ These observations suggest that the interlayer‐induced microstructural changes in the channel layer could, at least partially, account for the degraded performance observed in OFETs with thicker PEOz layers.

Another plausible reason for the aforementioned performance degradation seen in devices incorporating thicker PEOz is its electrically resistive nature. Evidence of the latter is found in the transistors’ transfer characteristics in Figure 2b, where the channel current is found to initially increase (PEOz (1) and PEOz (2)) and then decrease (PEOz (3) and PEOz (4)) as the thickness of the PEOz layer increases further. The same trend is observed in the contact resistance (Figure 2f), where R_C_W initially drops (PEOz (2)), as compared to PEOz (0) devices, and then increases again (PEOz (4)). On the basis of these results we conclude that there is an optimal thickness for the PEOz layer at which the work function of the S/D electrodes reduces enough while the resistivity of the interlayer remains relatively low so carrier injection becomes optimal. The latter condition, which here appears to be reached in PEOz (2)‐based transistors, leads to an all‐round device performance improvement, including the observed higher μ_sat_ and lower V_TH_ (Figure 2c,d).

**Figure 4:**
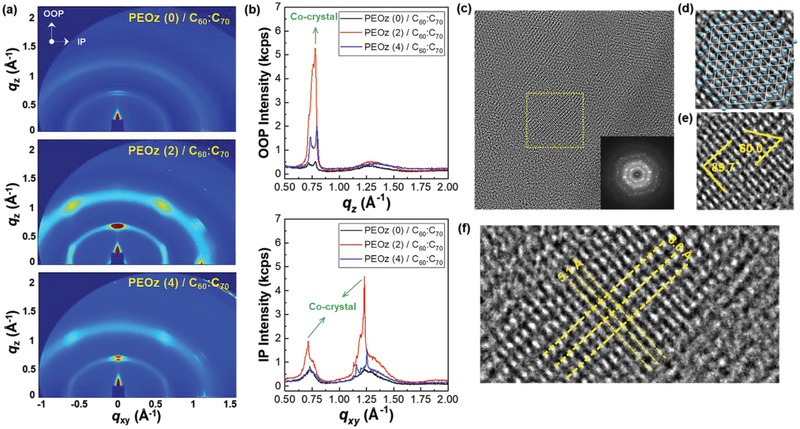
a) 2D GIXD images and b) 1D profiles for PEOz (0), PEOz (2), and PEOz (4) C_60_:C_70_ blend coated thin films. c–f) HR‐TEM images for C_60_:C_70_ co‐crystals on the PEOz (2) interlayer. Fourier transform patterns for the HR‐TEM image are shown in (c). Enlarged TEM images of the yellow square are shown in (d) and (e) which respectively show superimposed, a projection of the fcc crystal model along the [112] zone axis and the expected co‐crystal angles. f) *d*‐spacings for the fcc crystal model projected onto an enlarged part of the real space image.

Further studies were undertaken using synchrotron GIXD and HR‐TEM to more fully characterize the C_60_:C_70_ blend film nanostructure. As shown in **Figure** **4**a, isotropic and relatively broad diffraction rings were observed in the 2D GIXD image for C_60_:C_70_ blend films without a PEOz interlayer, indicating the presence of randomly oriented and relatively small crystallites; this is consistent with the corresponding TEM images (Figure S10a, Supporting Information). Strikingly, the GIXRD for C_60_:C_70_ blend films deposited on PEOz (2) interlayers display the features of highly crystalline regions with intense diffraction spots in both the in‐(IP) and out‐of‐plane (OOP) directions and subsidiary spots in two off axis directions. In addition, whilst isotropic diffraction rings are still observed in the background, they are narrower than for the PEOz (0) samples. For the PEOz (4) C_60_:C_70_ blend film samples the crystalline spots are still present but significantly diminished, showing a non‐monotonic variation in crystalline order that mirrors the non‐monotonic behavior of the transistor characteristics. 1D intensity line profiles along the OOP and IP directions (Figure 4b) reveal additional detail and confirm that the peak intensities for PEOz (2) C_60_:C_70_ samples are significantly higher than those for PEOz (4) and both higher than for PEOz (0) samples.

It has been reported that face‐centered‐cubic (fcc) and hexagonal‐close‐packed (hcp) structures are favored for C_60_ and C_70_ fullerenes, respectively, when separately deposited under ambient conditions.^[^ ^52^, ^53^, ^54^, ^55^, ^56^, ^57^ ^]^ Here, we observe formation of a well‐ordered C_60_:C_70_ co‐crystal structure, with pronounced diffraction peaks at *q*_z_ = 0.779 Å^−1^ and *q*_xy_ = 0.713 and 0.123 Å^−1^, corresponding to *d*‐spacings of 8.07, 8.81, and 5.10 Å, respectively which do not correspond to the fcc and hcp crystal phases for C_60_ and C_70_ fullerenes, respectively (see Figure S11, Supporting Information). The *q* and *d* values are related by *q* = (4π/λ) × sin(θ) = 2π/*d* where *d*, λ, and θ are the *d*‐spacing, incident X‐ray wavelength, and half of the scattering angle, respectively.

The highly ordered crystalline structure of the PEOz(2)/C_60_:C_70_ blend film is supported by the Fourier‐transform diffraction patterns of HR‐TEM images, as shown in Figure 4c. Real space analysis of the HR‐TEM images further confirms the fcc phase co‐crystal structure for the PEOz(2)/C_60_:C_70_ blend film. Direct comparison is made in Figure 4d with the superimposed 2D‐projection of the fcc crystal model along the [112] zone axis and the measured angles (89.7° and 60.0°) and distances (5.1 Å and 8.0 Å) in the TEM image match the expected values. We note that C_60_:C_70_ co‐crystals have been discussed previously in the context of exotic flower‐shaped crystal morphologies obtained when crystallization is driven by the addition of a poor solvent (ethanol) to mesitylene solutions containing both C_60_ and C_70_.^[^ ^58^ ^]^ These co‐crystals differ significantly from the present work in that they contain a fraction of mesitylene in addition to the C_60_ and C_70_ molecules and were not produced in a device‐compatible thin film format but rather as 3D‐structured artifacts.

### 2.4 Spatial Uniformity of OFET Performance

The distribution of μ_sat_ values was mapped for 10 × 4 arrays of PEOz (0) and PEOz (2) C_60_:C_70_ OFETs (see Figure S12, Supporting Information). PEOz (2) transistors exhibited ≈ 32 times higher average μ_sat_ = 1.6 ± 0.4 cm^2^ V^−1^ s^−1^, compared to transistors with no PEOz interlayer where the average μ_sat_ = 0.05 ± 0.04 cm^2^ V^−1^ s^−1^. In addition, the spread of mobility values is much narrower for the PEOz (2) transistors, as illustrated by the mobility color coding in Figure S12, Supporting Information. Inclusion of the PEOz (2) interlayer promotes excellent spatial uniformity in addition to the enhanced mobility. From a practical standpoint, such uniformity is highly beneficial for reproducibility and, together with the observed shelf‐life and ambient performance, is encouraging for applications. Furthermore, the beneficial effects of the PEOz interlayer appear generic and may well extend to other high performing n‐type organic semiconductors and OFETs.

## 3 Conclusion

PEOz has been demonstrated as an effective interfacial dipole layer to improve the performance of n‐type OFETs across a wide range of fullerenes and conjugated polymers. The Al/Au S–D electrode work function reduction achieved by deposition of PEOz films that adopt a nanodot structure has a significant impact on the charge injection/transport in these organic semiconductors. All devices with a PEOz (2) interlayer exhibited improved OFET performance in terms of their *V*_TH_ and μ_sat_ values. In particular, the maximum μ_sat_ value for C_60_:C_70_ OFETs with a PEOz (2) interlayer was 18.6 times larger than for C_60_:C_70_ OFETs with no interlayer. The non‐monotonic change in μ_sat_ contrasts with a monotonic reduction in work function and points to additional factors, such as the resistivity of the PEOz interlayer, playing an important role. For PEOz (2) transistors a fcc co‐crystal structure is observed for the C_60_:C_70_ blend film whereas this co‐crystal structure does not arise for other PEOz thicknesses. It is expected, therefore, that this novel packing arrangement supports the optimal mobility. The PEOz (2) interlayer also supports high spatial uniformity across an array of simultaneously fabricated transistors with a relatively tight distribution of mobility values. The results clearly demonstrate the ability to tune transistor performance through control of the semiconductor nanostructure, using solution deposited PEOz layers. Future studies will be needed to discern more fully the crystal growth mechanisms for solution‐processed and thermally evaporated fullerenes and the circumstances that promote co‐crystal formation and optimisation. Together with the development of low‐operating‐voltage devices based on thin (<100 nm) or high‐*k* (>10) gate dielectric layers it should then be possible to further enhance the C_60_:C_70_ OFET performance toward practical application.

## 4 Experimental Section

### Materials and Solutions

PEOz (weight‐average molecular weight ($\bar{M_{W}}$) = 50 kDa, polydispersity index (PDI) = 3–4) was supplied by Sigma Aldrich (USA) and used without further purification. The PEOz methanol solutions were prepared with concentrations from 1 to 4 mg mL^−1^ in 1 mg mL^−1^ increments. C_60_, C_70_, PC_61_BM, PC_71_BM, and ICBA (>99%) were purchased from Solenne BV (The Netherlands). P(NDI2OD‐T2) (*M*_w_ = 25–50 kDa, PDI = 1.5–3.5) was supplied by Polyera (USA) and P(DPPT‐TT) (*M*_w_ = 70 kDa, PDI = 3.1) was synthesized, as previously reported.^[^ ^59^ ^]^ CYTOP was used as received from Asahi Glass (Japan). The blend solution of C_60_ and C_70_ fullerenes (C_60_:C_70_ = 1:1 by weight) was prepared in dichlorobenzene at a solid concentration of 20 mg mL^−1^. PC_61_BM, PC_71_BM, and ICBA solutions were dissolved in chlorobenzene at a solid concentration of 15 mg mL^−1^. P(NDI2OD‐T2) and P(DPPT‐TT) solutions were prepared using chlorobenzene at a solid concentration of 5 mg mL^−1^. Each of these solutions was vigorously stirred on a magnetic stirring plate at 60 °C for 24 h before spin coating.

### Thin Film and Device Fabrication

Glass substrates were sequentially cleaned with acetone and isopropyl alcohol in ultrasonic baths for 10 min each. After being dried with flowing nitrogen, they were further treated with UV‐zone exposure for 20 min. The 5 nm‐thickness Al S–D electrode layers were first deposited onto borosilicate float glass substrates by thermal evaporation through a shadow mask and then overcoated with 25 nm‐thickness Au electrode layers using the same shadow mask. The mask specified channel width was 1000 µm, and structures with channel lengths of 30, 40, 50, and 80 µm were fabricated. The PC_61_BM, PC_71_BM, ICBA, and C_60_:C_70_ binary blend fullerene solutions were spin‐coated on top of the Al/Au electrode‐patterned glass substrates and annealed at 110 °C for 10 min in a nitrogen‐filled glove box. P(NDI2OD‐T2) and P(DPPT‐TT) conjugated polymer solutions were also spin‐coated on Al/Au‐electrode‐patterned glass substrates and annealed at 120 °C and then 150 °C for 10 min each in a nitrogen‐filled glove box. Next, 900 nm‐thick CYTOP (capacitance = 2.1 nF cm^−2^) was spin‐coated on top of the organic semiconductor layer and annealed at 60 °C for 3 h. The CYTOP gate dielectric layer helps to reduce energetic disorder at the interface between the organic semiconductor and gate dielectric and serves to passivate the device from the effects of moisture and oxygen exposure. Finally, 60 nm‐thick Al films were thermally evaporated using a shadow mask to define the gate electrodes. A separate set of devices was also constructed with PEOz interlayers interposed between the S–D electrodes/glass substrates and the semiconductor film. PEOz films were spin‐coated from 1, 2, 3, and 4 mg mL^−1^ methanol solutions. All other processing steps were as used for the structures without PEOz. For the preparation of TEM samples, C_60_:C_70_ blend films spin‐coated on PEOZ‐coated glass substrates were released by etching the glass substrates in 5 mM hydrofluoric acid solution. The resulting floating films were collected on TEM copper mesh grids without a carbon film support and dried in vacuo for 24 h.

### Thin Film and Device Measurements

The film thickness was measured using a surface profilometer (Tencor Instruments, α‐Step 200). UPS measurements were performed using an ultrahigh vacuum ESCALAB 250Xi system (Thermo Scientific) at a base pressure of ≈ 1 × 10^−9^ mbar. The photon excitation energy was 21.2 eV (He (I)) with an applied sample bias of −5 V. The work functions were also examined using an APS01 (KP Technology) KP system with a 2 mm diameter gold tip. Measurements were performed under ambient conditions and the work function values were calibrated against highly oriented pyrolytic graphite. The device characteristics were analyzed using a two‐channel semiconductor analyzer (Agilent B2986) inside a nitrogen‐filled glovebox. The surface morphology of the thin films was measured using AFM (Bruker, Nanoscope V Multimode 8). The X‐ray analysis of thin films was performed using synchrotron‐radiation GIXD measurements, with wavelength, λ = 1.2435 Å, at an incidence angle of 0.14° (3C, SAXS I beamline, Pohang Accelerator Laboratory, Korea). HR‐TEM images and electron diffraction patterns were obtained using Titan G2 ChemiSTEM Cs Probe (FEI Company) and field‐emission gun (FEG)‐TEM (JEOL 3000F) microscopes operated at 200 and 300 kV accelerating voltages, respectively.

## Conflict of Interest

The authors declare no conflict of interest.

## Supporting information

Supporting Information

## Data Availability

Research data are not shared.
