# Supplementary material for: Significant Performance Improvement in n‐Channel Organic Field‐Effect Transistors with C60:C70 Co‐Crystals Induced by Poly(2‐ethyl‐2‐oxazoline) Nanodots
Source: Adv Mater. 2021 Jun 24;33(31):2100421. doi: 10.1002/adma.202100421 (PMC11468551; doi:10.1002/adma.202100421)
Supplement: Supplementary file 1 — Supporting Information [file ADMA-33-2100421-s001.pdf]

© 2021 Wiley-VCH GmbH

ADVANCED MATERIALS

Supporting Information

for *Adv. Mater.,* DOI: 10.1002/adma.202100421

Significant Performance Improvement in n-Channel Organic Field-Effect Transistors with C_{60}:C_{70} Co-Crystals Induced by Poly(2-ethyl-2-oxazoline) Nanodots

Sungho Nam, Dongyoon Khim, Gerardo T. Martinez, Aakash Varambhia, Peter D. Nellist, Youngkyoo Kim, Thomas D. Anthopoulos,* and Donal D. C. Bradley**

Copyright Wiley-VCH GmbH, 2021.

Supporting Information

Significant Performance Improvement in n-Channel Organic Field-Effect Transistors with C_{60}:C_{70} Co-Crystals Induced by Poly(2-ethyl-2-oxazoline) Nanodots

S. Nam, D. Khim, G. T. Martinez, A. Varambhia, P. D. Nellist, Y. Kim, T. D. Anthopoulos*, and D. D. C. Bradley**

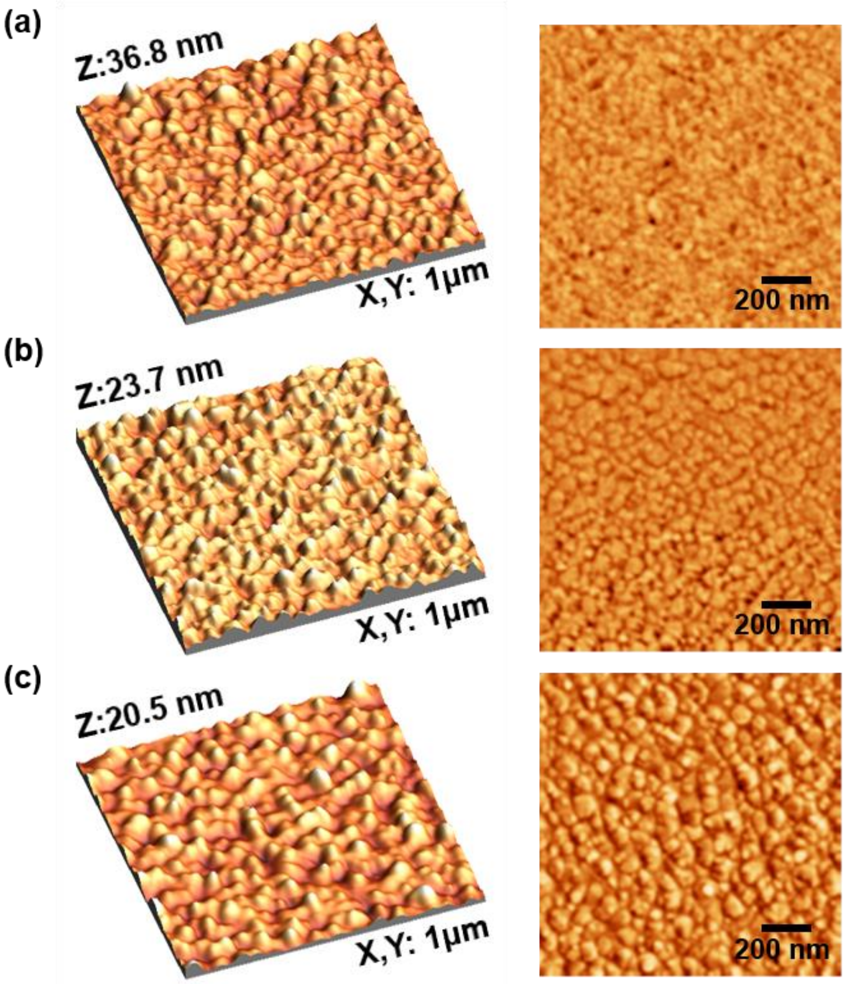

Figure S1. 3D height-mode (left) and phase-mode (right) AFM images: (a) glass/Al/Au/PEOz (0), (b) glass/Al/Au/PEOz (2), and (c) glass/Al/Au, PEOz (4).

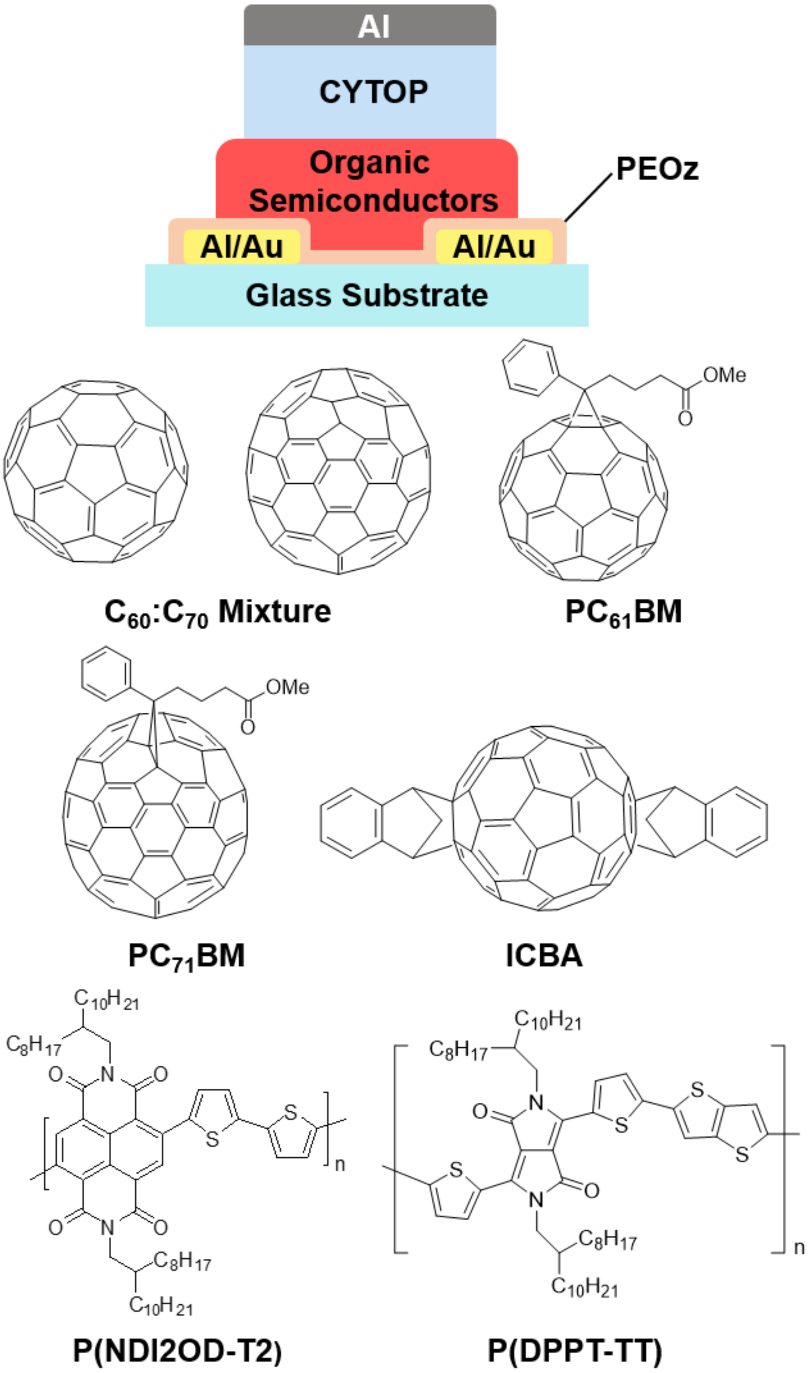

Figure S2. Schematic illustration for the OFET structure with the PEOz layer between the source-drain (Al/Au) electrodes and the organic semiconductor layers (see below for the chemical structure of fullerene derivatives and polymer semiconductors used as channel layers in this study).

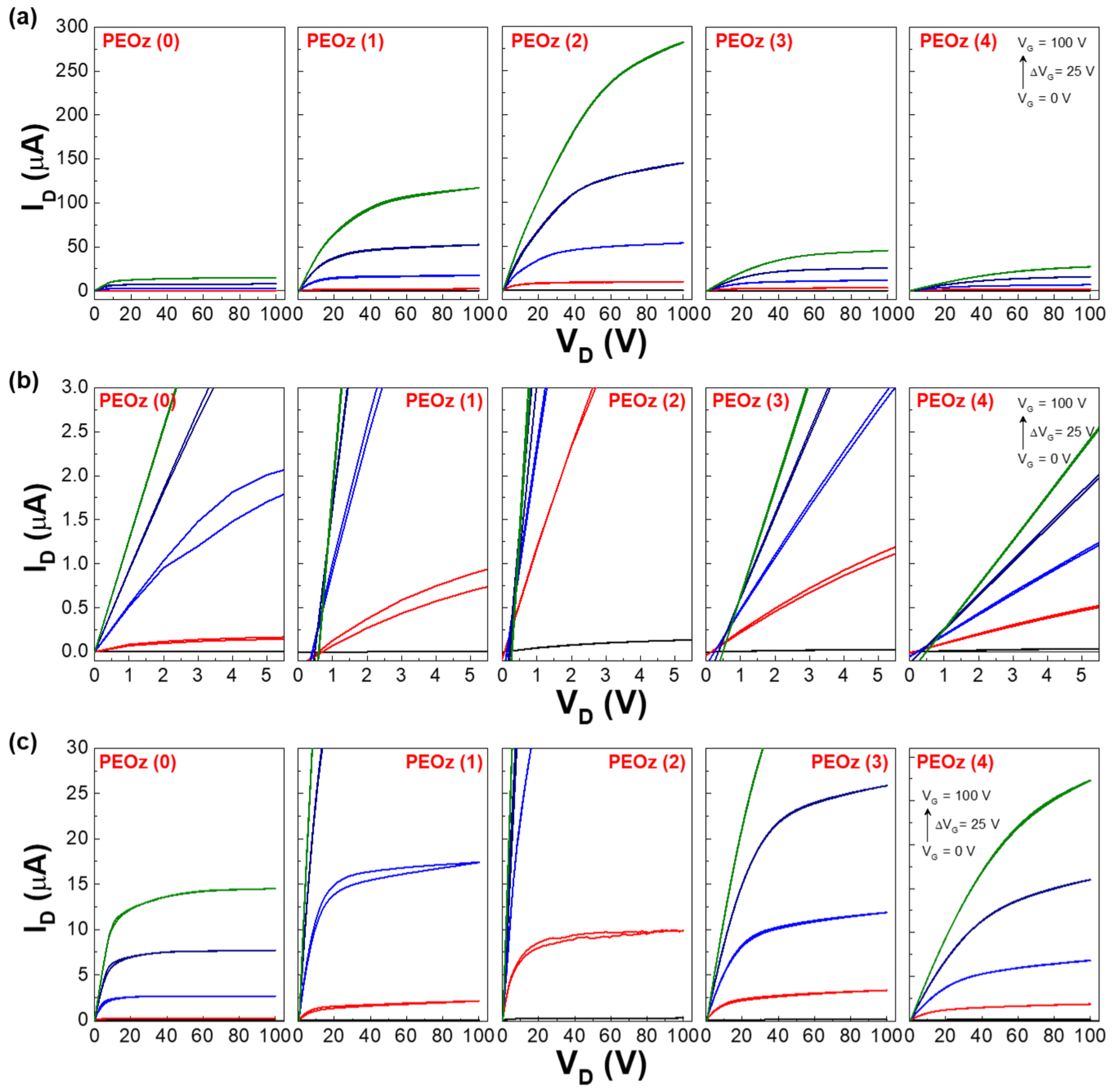

Figure S3. (a) Output characteristics for the C_{60}:C_{70} OFETs with the corresponding PEOz solution concentration (PEOz (0) = 0 mg/ml ~ PEOz (4) = 4 mg/ml). Replotted output curves from (a): (b) low V_D ($\leq$ 5.5 V) and (c) high V_D ($\leq$100 V)

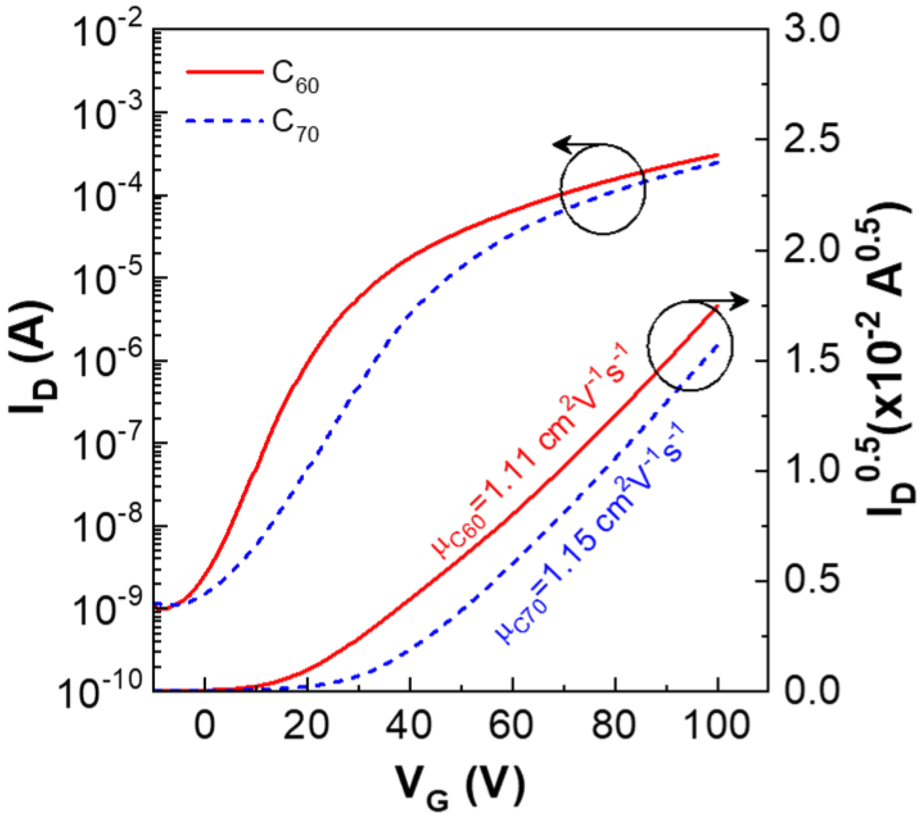

Figure S4. Transfer characteristics at V_D = 100 V for the pristine C_{60} or C_{70} fullerene transistors with the PEOz (2) interlayer.

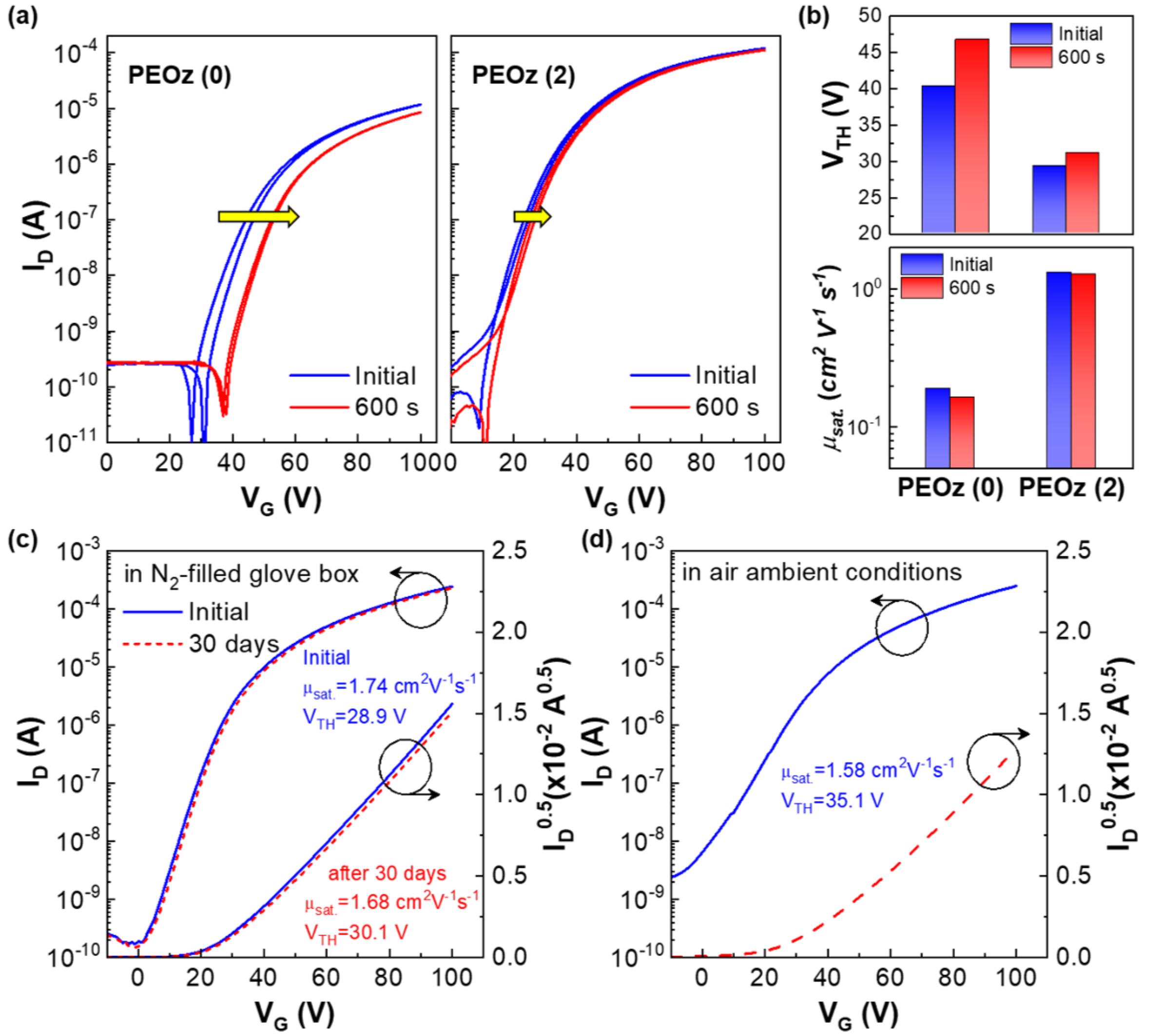

Figure S5. (a) Transfer curves for C_{60}:C_{70} transistors with the PEOz (0) and PEOz (2) interlayers both before and after 600s bias stress at V_D and V_G = 30 V, and (b) the resulting threshold voltage (V_{TH}) and field-effect mobility ($\mu_{sat.}$) values in the saturation region. (c,d) Transfer curves at V_D = 100 V for C_{60}:C_{70} transistors with the PEOz (2) interlayers before and after 30 days in N_2-filled glove box (c) and stored in N_2-filled glove box before measurement and measured in air ambient conditions (d).

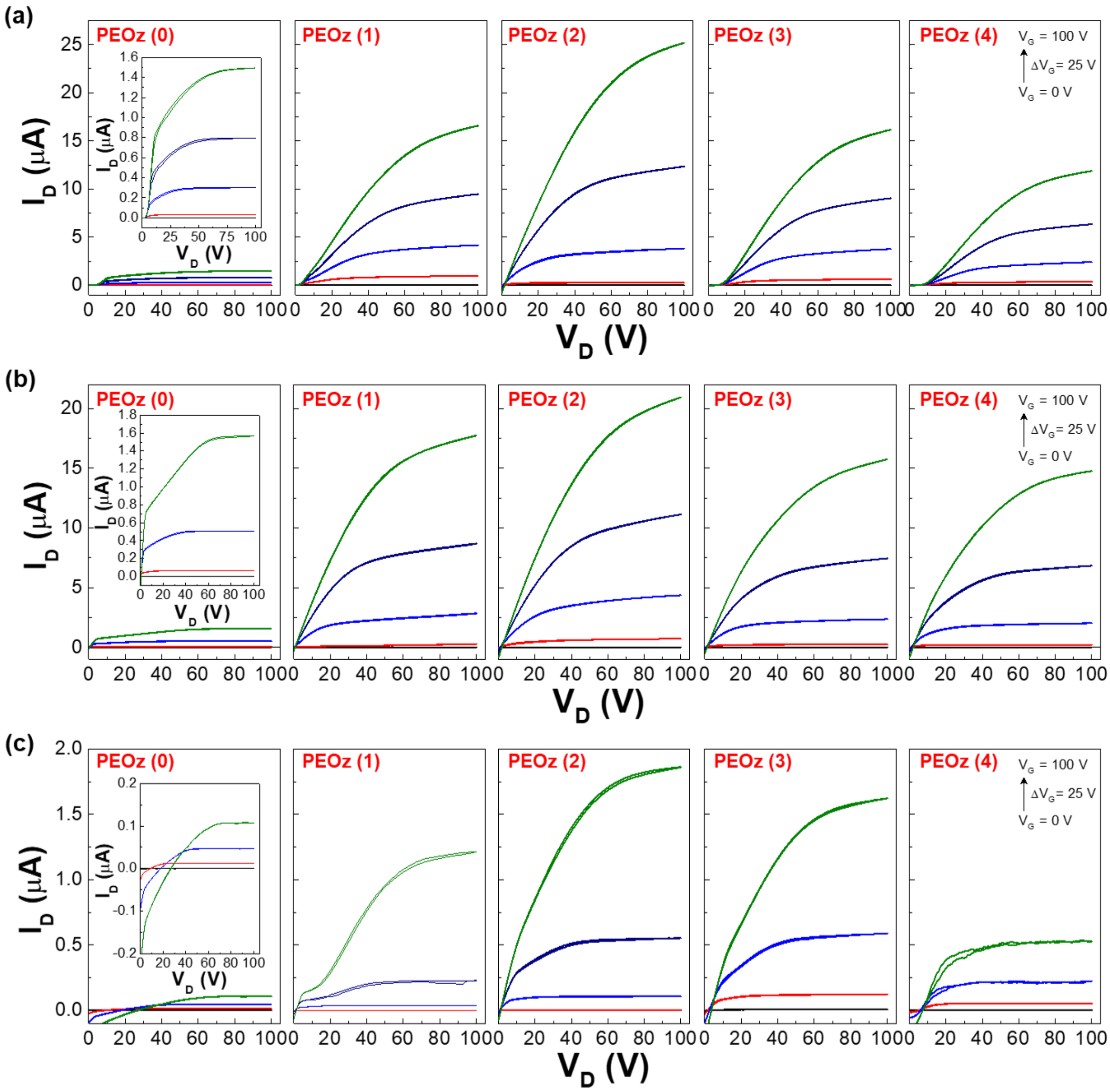

Figure S6. Output characteristics for the OFETs with the PEOz layers according to the PEOz solution concentration: (a) $PC_{61}BM$, (b) $PC_{71}BM$, and (c) ICBA.

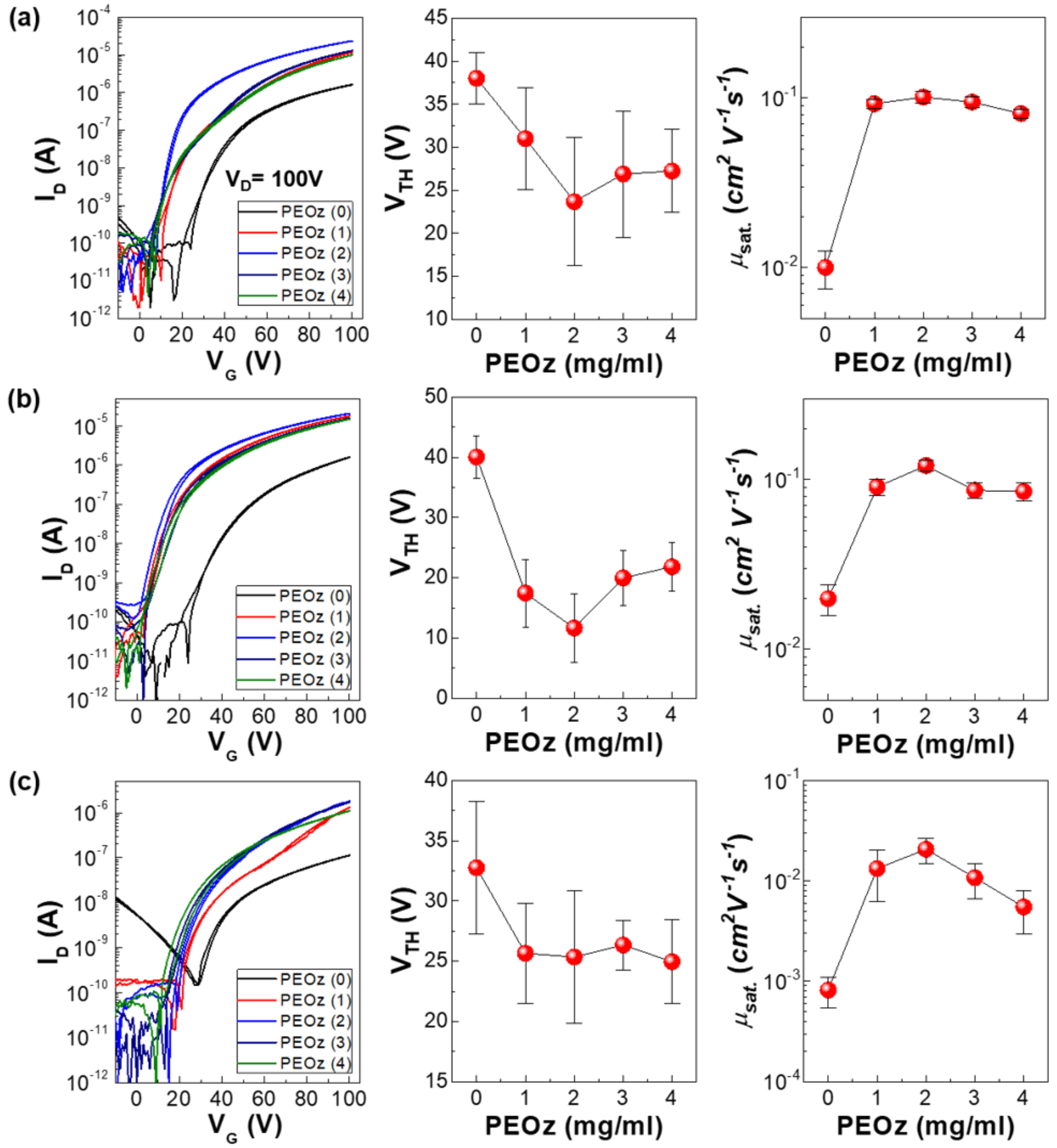

Figure S7. Transfer characteristics at V_D = 100 V for the OFETs with the PEOz layers according to the PEOz solution concentration (see threshold voltage (V_{TH}) and field-effect mobility ($\mu_{sat.}$) in the saturation region as a function of the PEOz solution concentration): (a) $PC_{61}BM$, (b) $PC_{71}BM$, and (c) ICBA.

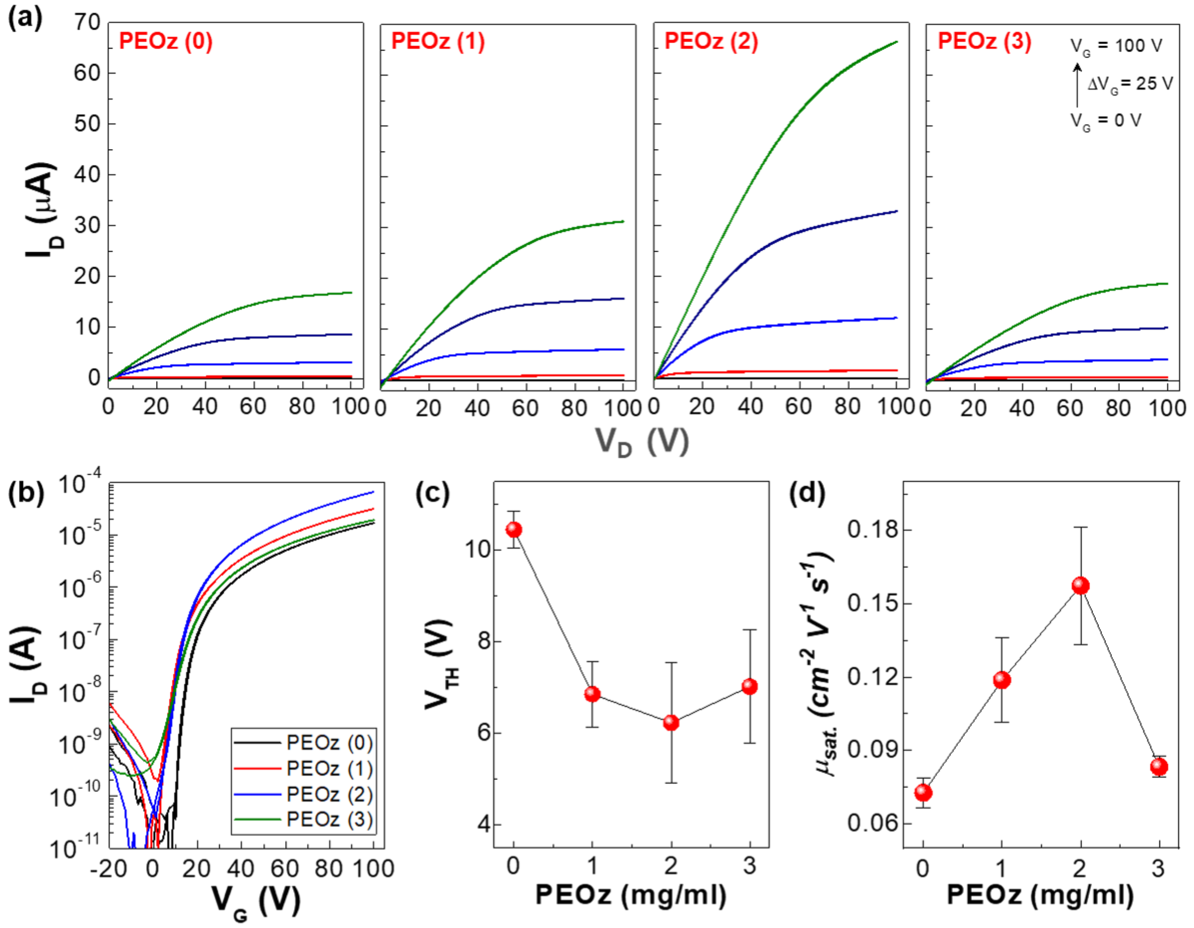

Figure S8. (a) Output and (b) transfer characteristics at V_D = 100 V for P(NDI2OD-T2) OFETs at the corresponding PEOz solution concentration. (c) Threshold voltage (V_{TH}) and (d) field-effect mobility ($\mu_{sat.}$) in the saturation region as a function of the PEOz solution concentration.

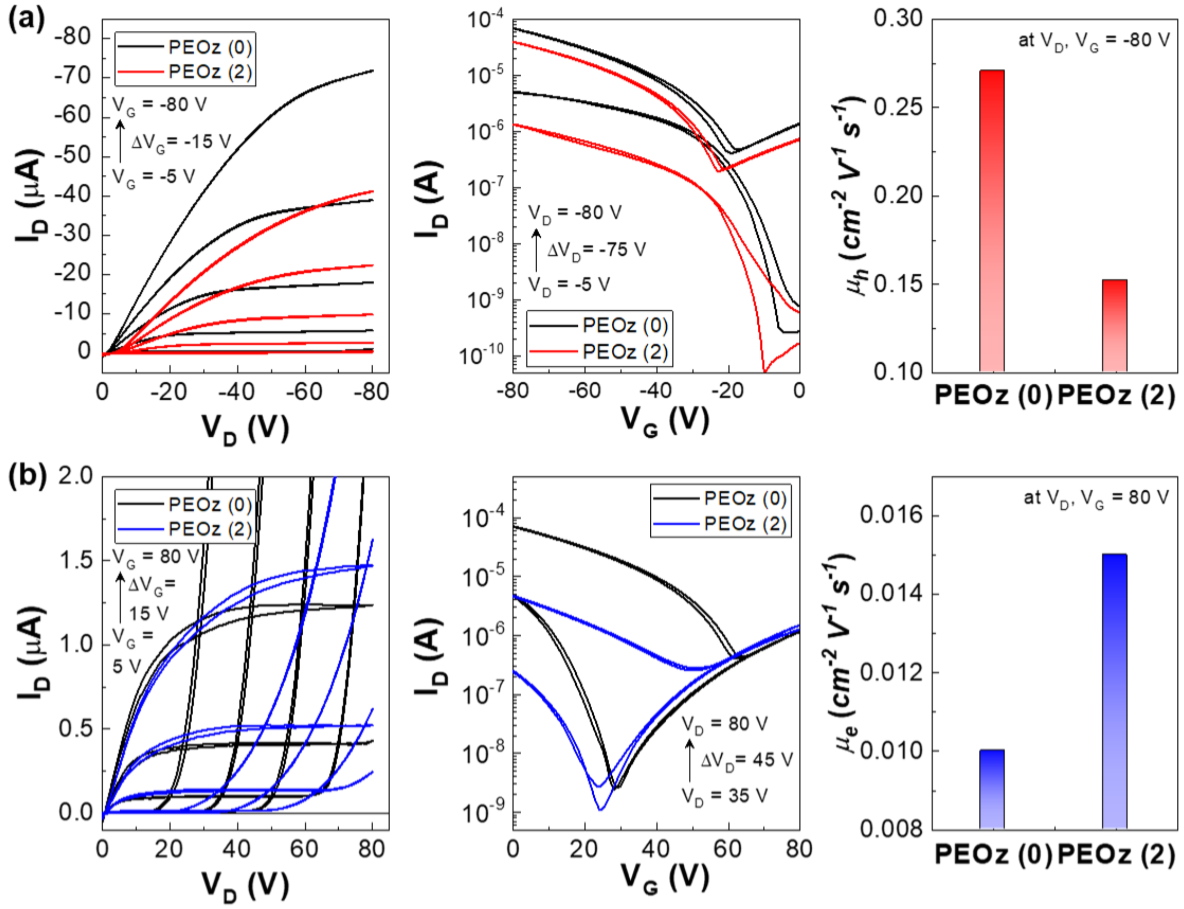

Figure S9. Output and transfer characteristics for P(DPPT-TT) OFETs with the PEOz(0) and PEOz(2) interlayers (see the right panels for the field-effect mobility for holes (μ_h) and electrons (μ_e) in the saturation region): (a) p-type (V_D and $V_G \leq 0$ V) and (b) n-type (V_D and $V_G \geq 0$ V) region.

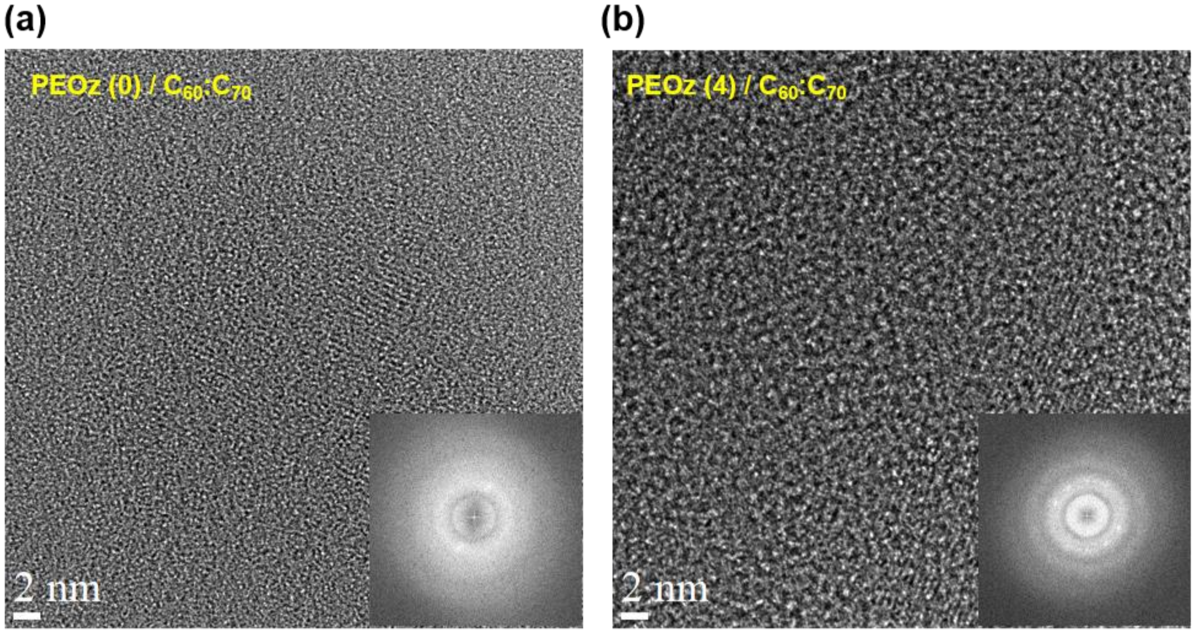

Figure S10. HR-TEM images and Fourier transform patterns for C_{60}:C_{70} (10:10 by weight) thin films: (a) PEOz (0) and (b) PEOz (4) interlayers.

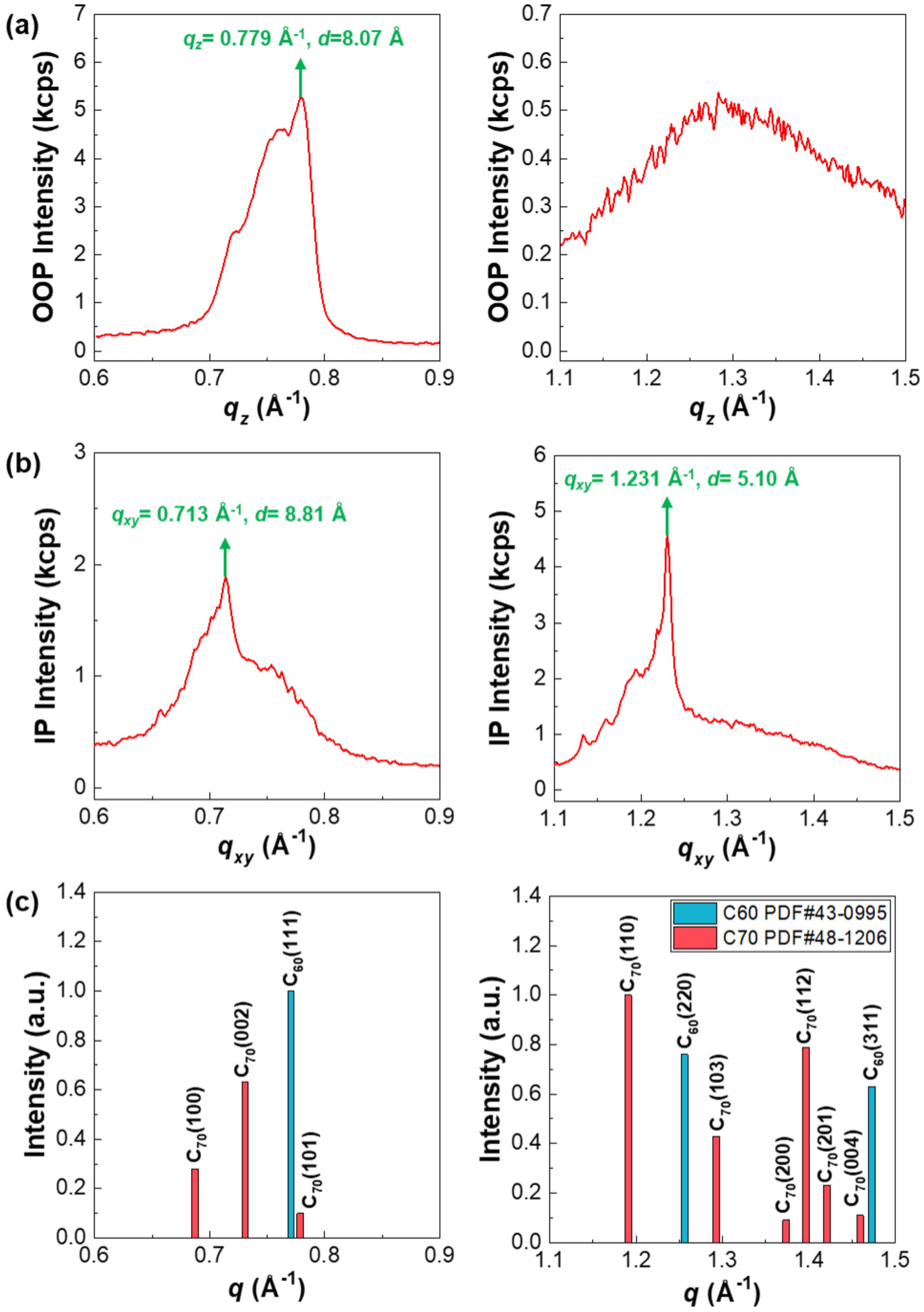

Figure S11. 1D profiles for C_{60}:C_{70} co-crystals on a PEOz (2) interlayer in the (a) out-of-plane (OOP) and (b) in-plane (IP) directions. (c) Joint committee on powder diffraction

standards- international centre for diffraction data (JCPDS-ICDD) powder diffraction file (PDF) for the C_{60} and C_{70} standard [C_{60} for PDF#43-0995 and C_{70} for PDF#48-1206]

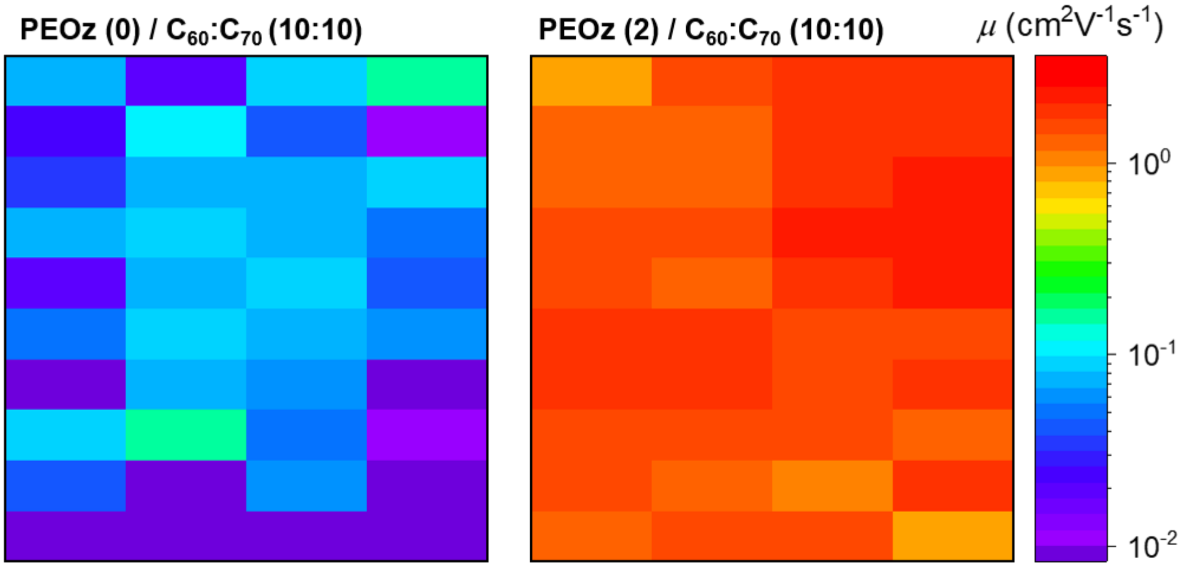

Figure S12. Spatial distributions of μ_{sat} values (colour-coded) for 40 (10 × 4) OFETs without any PEOz layer (left panel) and with the PEOz (2) interlayer (right panel).

Table S1. Summary of device parameters as a function of the PEOz solution concentration for the OFETs with soluble fullerene and conjugated polymer semiconductors.

**Material**	**PEOz solution concentration**	**Charge Carrier**	**V_{TH} (V)**	**$\mu_{sat.}$ ($cm^{-2}\ V^{-1}\ s^{-1}$)**
C_{60}:C_{70}	0	Electron	31.54 ±5.94	0.11 ±0.05
	1		30.21 ±5.12	0.71 ±0.16
	2		27.45 ±4.32	2.05 ±0.11
	3		28.78 ±5.51	0.45 ±0.14
	4		28.91 ±3.79	0.15 ±0.01
C_{60}	2	Electron	24.45 ±1.79	1.11 ±0.13
C_{70}	2	Electron	36.42 ±2.13	1.15 ±0.12
$PC_{61}BM$	0	Electron	38.01 ±2.99	0.01 ±0.002
	1		31.01 ±5.94	0.09 ±0.005
	2		23.67 ±7.45	0.10 ±0.008
	3		26.87 ±7.34	0.095 ±0.007
	4		27.22 ±4.81	0.081 ±0.004
$PC_{71}BM$	0	Electron	40.04 ±3.52	0.019 ±0.004
	1		17.45 ±5.61	0.091 ±0.010
	2		11.65 ±5.69	0.121 ±0.008
	3		19.97 ±4.57	0.087 ±0.009
	4		21.83 ±4.06	0.085 ±0.010
ICBA	0	Electron	32.75 ±5.52	0.001 ±0.0002
	1		25.65 ±4.17	0.013 ±0.007
	2		25.34 ±5.48	0.021 ±0.006
	3		26.34 ±2.05	0.011 ±0.004
	4		24.95 ±3.46	0.005 ±0.002
P(NDI2OD-T2)	0	Electron	10.45 ±0.40	0.072 ±0.006
	1		6.86 ±0.72	0.120 ±0.017
	2		6.23 ±1.32	0.160 ±0.024
	3		7.02 ±1.24	0.083 ±0.004
P(DPPT-TT)	0	Hole	-24.79 ±2.39	0.385 ±0.047
		Electron	45.22 ±3.21	0.016 ±0.002
	2	Hole	-25.35 ±3.09	0.215 ±0.005
		electron	42.68 ±3.47	0.019 ±0.001
